# Development of a Dual Reporter System to Simultaneously Visualize Ca^2+^ Signals and AMPK Activity

**DOI:** 10.1021/acssensors.4c01058

**Published:** 2024-08-21

**Authors:** Yusuf C. Erdoğan, Johannes Pilic, Benjamin Gottschalk, Esra N. Yiğit, Asal G. Zaki, Gürkan Öztürk, Emrah Eroğlu, Begüm Okutan, Nicole G. Sommer, Annelie M. Weinberg, Rainer Schindl, Wolfgang F. Graier, Roland Malli

**Affiliations:** † Gottfried Schatz Research Center, Molecular Biology and Biochemistry, Medical University of Graz, Neue Stiftingtalstraße 6, Graz 8010, Austria; ‡ BioTechMed Graz, Mozartgasse 12/2, Graz 8010, Austria; § Regenerative and Restorative Medicine Research Center (REMER), Research Institute for Health Sciences and Technologies (SABITA), Istanbul Medipol University, Istanbul 34810, Turkey; ∥ Department of Physiology, International School of Medicine, İstanbul Medipol University, İstanbul 34810, Türkiye; ⊥ Department of Orthopedics and Traumatology, Medical University of Graz, Auenbruggerplatz 5, Graz 8036, Austria; # Gottfried Schatz Research Center, Biophysics, Medical University of Graz, Neue Stiftingtalstrasse 6, Graz 8010, Austria; ¶ Center for Medical Research, Bioimaging, Medial University of Graz, Neue Stiftingtalstrasse 6, Graz 8010, Austria

**Keywords:** AMPK reporter, Ca^2+^ biosensor, fluorescence microscopy, phase separation-based activity reporter of kinase, SPARK, GCaMP, multiplexing

## Abstract

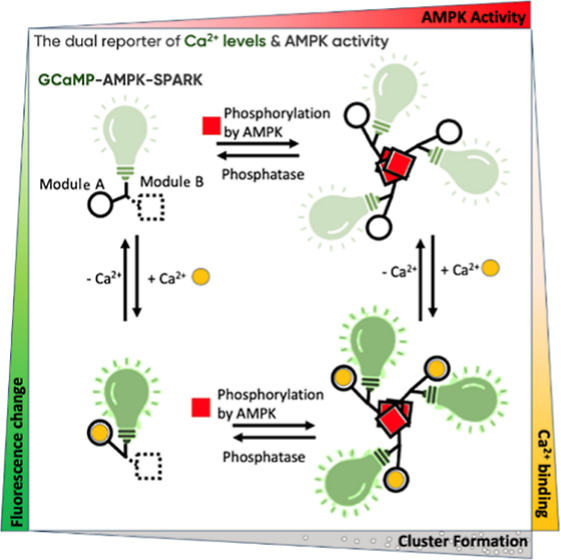

In this study, we introduce a new separation of phases-based activity reporter of kinase (SPARK) for AMP-activated kinase (AMPK), named AMPK-SPARK, which reports the AMPK activation by forming bright fluorescent clusters. Furthermore, we introduce a dual reporter system, named GCaMP-AMPK-SPARK, by incorporating a single-fluorescent protein (FP)-based Ca^2+^ biosensor, GCaMP6f, into our initial design, enabling simultaneous monitoring of Ca^2+^ levels and AMPK activity. This system offers the essential quality of information by single-channel fluorescence microscopy without the need for coexpression of different biosensors and elaborate filter layouts to overcome spectral limitations. We used AMPK-SPARK to map endogenous AMPK activity in different cell types and visualized the dynamics of AMPK activation in response to various stimuli. Using GCaMP-AMPK-SPARK, we revealed cell-to-cell heterogeneities in AMPK activation by Ca^2+^ mobilization. We anticipate that this dual reporter strategy can be employed to study the intricate interplays between different signaling networks and kinase activities.

Protein kinases play a crucial role in regulating vital cellular processes by orchestrating the phosphorylation of proteins, a widely recognized and prevalent form of posttranslational modification that profoundly impacts signal transduction, cell metabolism, cell cycle progression, and gene expression.^1^ Measuring kinase activity at the single-cell level helps capture cellular heterogeneity, reveal dynamic responses, and assess drug responses accurately. FP-based biosensor technologies enabled the development of tools to help monitor real-time kinase activities in live cells with a high spatiotemporal resolution.^2^

AMPK is a crucial regulator of energy homeostasis in eukaryotic cells.^3^ Various genetic AMPK biosensors have been developed based on FPs to study AMPK activity at the single-cell level.^4−8^ However, these sensors demonstrate limited fluorescence changes upon kinase activation. In this study, we introduce a novel separation of phases-based activity reporter (SPARK) for AMPK, named AMPK-SPARK. The phase separation-based kinase reporter approach, introduced initially for visualizing the activity of protein kinase A (PKA) and extracellular signal-regulated kinase (ERK), uses a different physical principle than the available single FP- and Förster Resonance Energy Transfer (FRET)-based kinase biosensors.^9^ SPARK allows a high dynamic range in terms of the fluorescent change upon kinase activation. Drawing an analogy, we employed an AMPK-specific substrate peptide, previously utilized in developing single FP- and FRET-based genetic AMPK reporters,^4,7^ to design AMPK-SPARK.

AMPK activation is a multifaceted process that involves various signaling pathways in response to cellular stresses. These stresses include energy-related challenges such as hypoxia, ischemia, and heat shock, where fluctuations in AMP/ATP ratios and fructose-1,6-bisphosphate (FBP) levels play pivotal roles in sensing energy stress by AMPK.^10,11^ Cells respond to increases in AMP/ADP to ATP ratio and decrease in FBP by activating AMPK. The activation cascade involves two essential upstream kinases, liver kinase B1 (LKB1) and calcium/calmodulin-dependent protein kinase 2 (CaMKK2).^12^ The direct phosphorylation of AMPK by CAMKK2, triggered by a rise in intracellular Ca^2+^ levels, thus adds another layer of complexity to the regulatory network of noncanonical AMPK activation. Deciphering Ca^2+^-mediated regulation of AMPK activity at the single-cell level would require coimaging spectrally distinct AMPK and Ca^2+^ biosensors. However, such coimaging may be challenging due to complex microscopy setups and spectral limitations. To circumvent these challenges, we built on a recent report that established signaling reporter islands (SiRIs) with clustered biosensors to monitor signaling networks, demonstrating that single FP-based biosensors remain functional within clusters.^13^ Motivated by these findings, we replaced the enhanced green FP (EGFP) in AMPK-SPARK with a single FP-based Ca^2+^ biosensor, GCaMP6f,^14^ to create a dual reporter capable of simultaneously tracking intracellular Ca^2+^ levels and AMPK activity. Named GCaMP-AMPK- SPARK, this dual reporter operates solely through one channel (GFP), effectively overcoming the spectral challenges. We demonstrate that imaging with GCaMP-AMPK-SPARK offers comprehensive insights into the nuanced regulation of AMPK activation by Ca^2+^, shedding light on the variability of Ca^2+^ signals in triggering AMPK activation.

## Results and Discussion

### Design of EGFP-Based AMPK-SPARK

**Figure 1:**
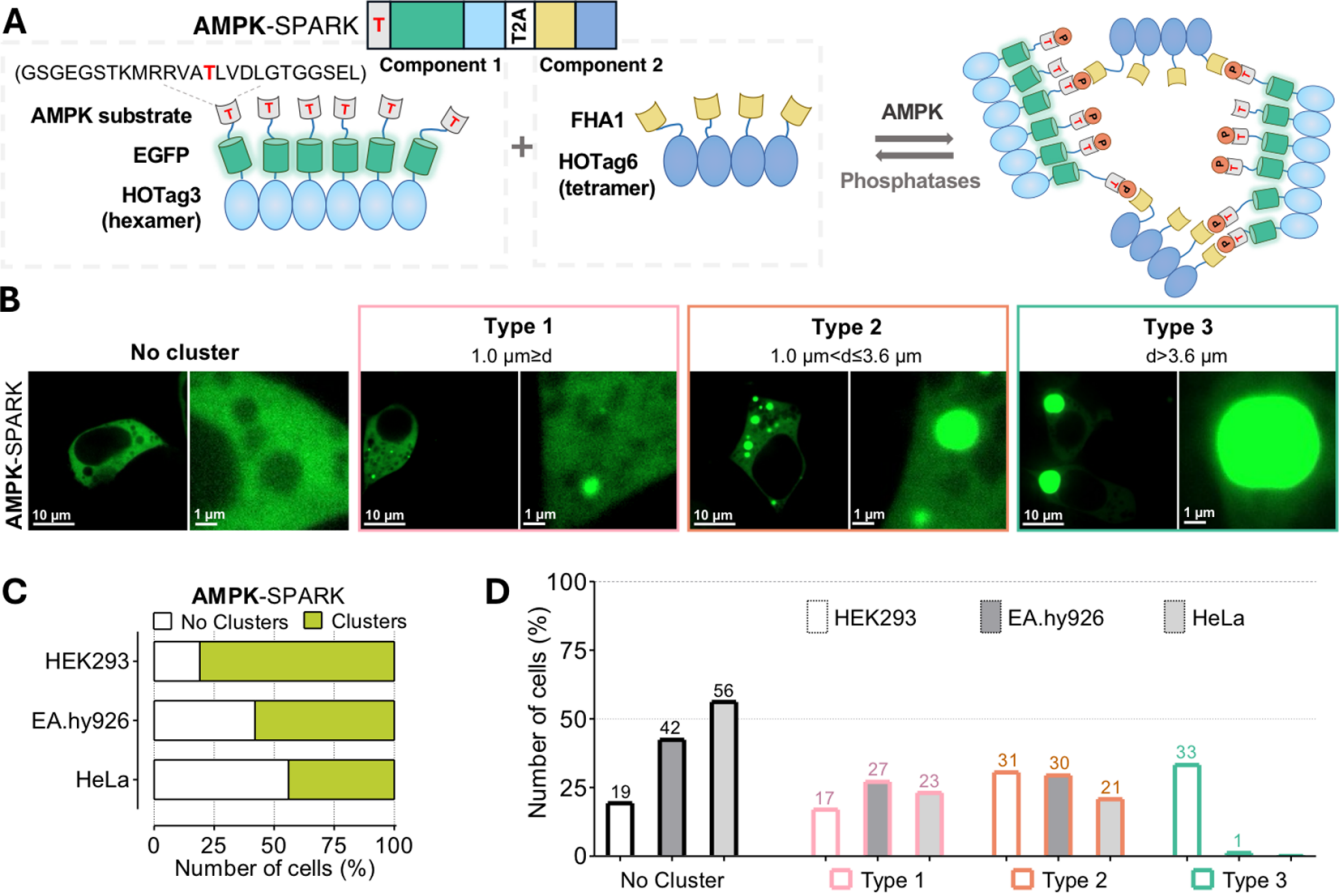
Mapping endogenous AMPK activity in different cell types using SPARK technology. (A) Schematic shows two components of AMPK-SPARK and their working principle. The first component consists of the AMPK-specific substrate sequence, EGFP, and hexameric HOTag3. The second component consists of the phosphobinder FHA1 sequence and tetrameric HOTag6. Upon AMPK activation, the two components cluster and form bright fluorescent droplets due to phase separation. Phosphatases work in the opposite direction, and they work to dissolve the clusters formed. (B) Confocal images show different types of HEK293D cells based on the diameter (d) of the largest cluster observed, transiently expressing AMPK-SPARK. (C) Bar graphs represent the proportion of cells for the presence of clusters in HEK293D (*n* = 3/259), EA.hy926 (*n* = 2/85), and HeLa (*n* = 3/130) upon transient expression of AMPK-SPARK. (D) Graph displays the type distribution with respect to cells described in panel C.

To achieve visualization of AMPK activity at the single-cell level with an enhanced dynamic range in terms of fluorescence change, we developed a multivalent interaction system based on phosphorylation-inducible phase separation, inspired by the recently established SPARK technology.^9^ Like other phase separation-based kinase reporters,^15,16^ AMPK-SPARK comprises two components (Figure 1A). In the first component, we fused the validated AMPK-specific substrate peptide sequence^4^ with an enhanced GFP (EGFP) and a coiled-coil domain (a hexameric tag homo-oligomeric tag 3, (HOTag3)), as shown in Figure 1A. In the second component of the reporter, we fused a phospho-threonine-binding domain, the forkhead-associated domain 1 (FHA1), to another coiled-coil domain, the tetrameric tag homo-oligomeric tag 6 (HOTag6, Figure 1A). A self-cleaving T2A sequence^17^ was used to link the two parts of the reporter to allow coexpression of both components. In cells where both components of SPARK-AMPK are present, activation of AMPK leads to multivalent protein–protein interactions, inducing phase separation resulting in intensely bright green fluorescent clusters, which, in turn, can reversibly dissolve upon activation of phosphatases^18^ (Figure 1A).

### AMPK-SPARK Reveals Cell-to-Cell Variations in Endogenous AMPK Activity

Next, we assessed the relative energy stress status of different cell lines in vitro using AMPK-SPARK. Therefore, we transfected human embryonic kidney (HEK) 293 cells, the well-known cervical cancer cell line, HeLa, and immortalized human umbilical vein endothelial cells, the EA.hy926 cells, with the new SPARK reporter system. The cells were then imaged 2 days after transfection. Interestingly, while some cells expressing AMPK-SPARK had uniform fluorescence, others had droplets of different sizes, inferring varying states of cellular energy stress among the cells within the same population under these conditions (Figure 1B–D). In HEK 293 cells expressing SPARK-AMPK, 81% displayed droplets; while in EA.hy926 and HeLa cells, the proportions of cells with droplets were 58 and 44%, respectively (Figure 1C). Notably, these proportions exhibited marked variations when PKA-SPARK, the SPARK-based PKA reporter,^9^ was expressed in the same cell lines (Figure S1A). Moreover, the phospho-null mutant reporter, AMPK^(*T*>A)^-SPARK, in which we substituted phospho-threonine with alanine to prevent phosphorylation by AMPK, demonstrated exclusively homogeneous fluorescence in the same cell lines (Figure S2). Based on these, we presumed that droplets formed upon AMPK-SPARK expression are intrinsic to AMPK activity and that they qualitatively offer insights into the on–off status of AMPK at the single-cell resolution. Exploiting the simple signal pattern of AMPK-SPARK, we classified three cases defined by the diameter of the largest droplet observed in a single cell. Suppose the diameter of the largest droplet observed is (*i*) smaller than 1.0 μm, (*ii*) between 1.0–3.6 μm, or (*iii*) larger than 3.6 μm, the cell was classified as type 1, type 2, or type 3, respectively (Figure 1B). Interestingly, we observed distinct distribution patterns of these AMPK-SPARK droplet types across the three cell types (Figure 1D), suggesting potential variations in endogenous AMPK and phosphatase activities or expression levels. Moreover, the observed heterogeneity within the same cell population and among different cell types may arise from the differential restriction of diffusion based on cell shape or macromolecular crowding levels between individual cells, which might impact both the kinase activities^19^ or the phase separation.^20^ Thus, the comparative interpretation of SPARK data should be done with caution. Furthermore, the expression of SPARK-PKA showed entirely different profiles of these types for the same cell lines (Figure S1B), suggesting inherent differences between endogenous AMPK and PKA activities.

Next, we investigated the impact of expression levels of AMPK-SPARK on cluster presence, count, volume, and sphericity (Figure S3) using high-resolution volumetric z-scan imaging of HeLa cells and compared the control and energy-stressed conditions. Independently of the cluster presence, we have cells over a wide spectrum of expression (Figure S3A). HeLa cells were pretreated with a glucose-free medium supplemented with 2-DG (2-Deoxy-d-glucose), a glucose analog that inhibits glycolysis and strongly reduces cellular ATP levels,^21,22^ to induce severe energy stress. Such energy stress yielded a higher cluster count (Figure S3B), total cluster volume (Figure S3C), and mean cluster volume (Figure S3D) compared to the control condition. We observed no significant difference in cluster sphericity under these two conditions (Figure S3E). Moreover, we did not find any correlations between expression levels and cluster counts or sphericity. However, under energy stress, we determined a moderate correlation between expression levels and cluster volume (Figure S3C,D). Although these results indicate that the expression level has a minimal impact on cluster formation and morphology, we recommend using cells with similar expression levels for comparative analyses. If possible, then normalization to the expression level is advised. Additionally, we think that SPARK technology should be primarily utilized for qualitative rather than quantitative investigations.

Previous studies reported that the dissociation of Mg^2+^ from ATP influences cellular energy metabolism and response to energy stress.^23,24^ However, the impact of the extracellular Mg^2+^ concentration on cellular energy stress remains unexplored. This aspect is particularly relevant in assessing the biodegradation of magnesium implants used in bone fixation surgeries.^25^ To investigate this, we employed AMPK-SPARK in EA.hy926 cells cultured with varying Mg^2+^ concentrations (Figure S4). Without adding Mg^2+^ to the culture medium, we observed an increase of 14% of endothelial cells showing no AMPK-SPARK clusters, indicating that a reduction in Mg^2+^ might lower cellular energy stress. AMP binding to the γ subunit of the AMPK facilitates activation of AMPK, which is counteracted by the presence of Mg^2+^-unbound free ATP.^26,27^ We think that reduced levels of Mg^2+^ (0 mM) may increase the levels of Mg^2+^-unbound free ATP, leading to lower AMPK activity. Surprisingly, high levels of extracellular Mg^2+^ (8 mM) showed no significant effect on the presence or size of AMPK-SPARK clusters compared to basal Mg^2+^ levels (0.8 mM) (Figure S4B). These findings suggest that high extracellular Mg^2+^ levels have a minimal impact on AMPK activity.

Delving deeper into the cells, we noted a distinct difference in the condensate morphology between AMPK-SPARK and PKA-SPARK clusters. Specifically, the clusters of AMPK-SPARK were predominantly spheroid in the three cell types tested (Figure S5A,B). In contrast, those of PKA-SPARK appeared to take on an amorphous structure in a considerable proportion across various cell types (Figure S5C,D). While the origin of these morphological differences remains unclear, it is intriguing to hypothesize that the morphology of SPARK clusters may indicate temporal variations in the interplay between the kinase and the phosphatase activities.^28^ Furthermore, we also identified notable differences in how SPARK clusters are spatially arranged within the cell, which may reflect the cellular organization of kinases to regulate signaling.^29^ While the spherical clusters of AMPK-SPARK are typically scattered over the cytosol (Figure S6A), the PKA-SPARK clusters were sporadically spotted at the subplasmalemmal space (Figure S6B) or around vesicular structures (Figure S6C,D). There is growing interest in investigating the biophysical principles and properties of biomolecular condensates.^30−32^ We think a more comprehensive examination of the morphology and subcellular localization of SPARK clusters may help gain deeper insights into the spatial and temporal dynamics of kinase activity alterations with this technology.

### AMPK-SPARK Dynamically Forms Clusters in Response to Canonical and Noncanonical Ways of Activation

**Figure 2:**
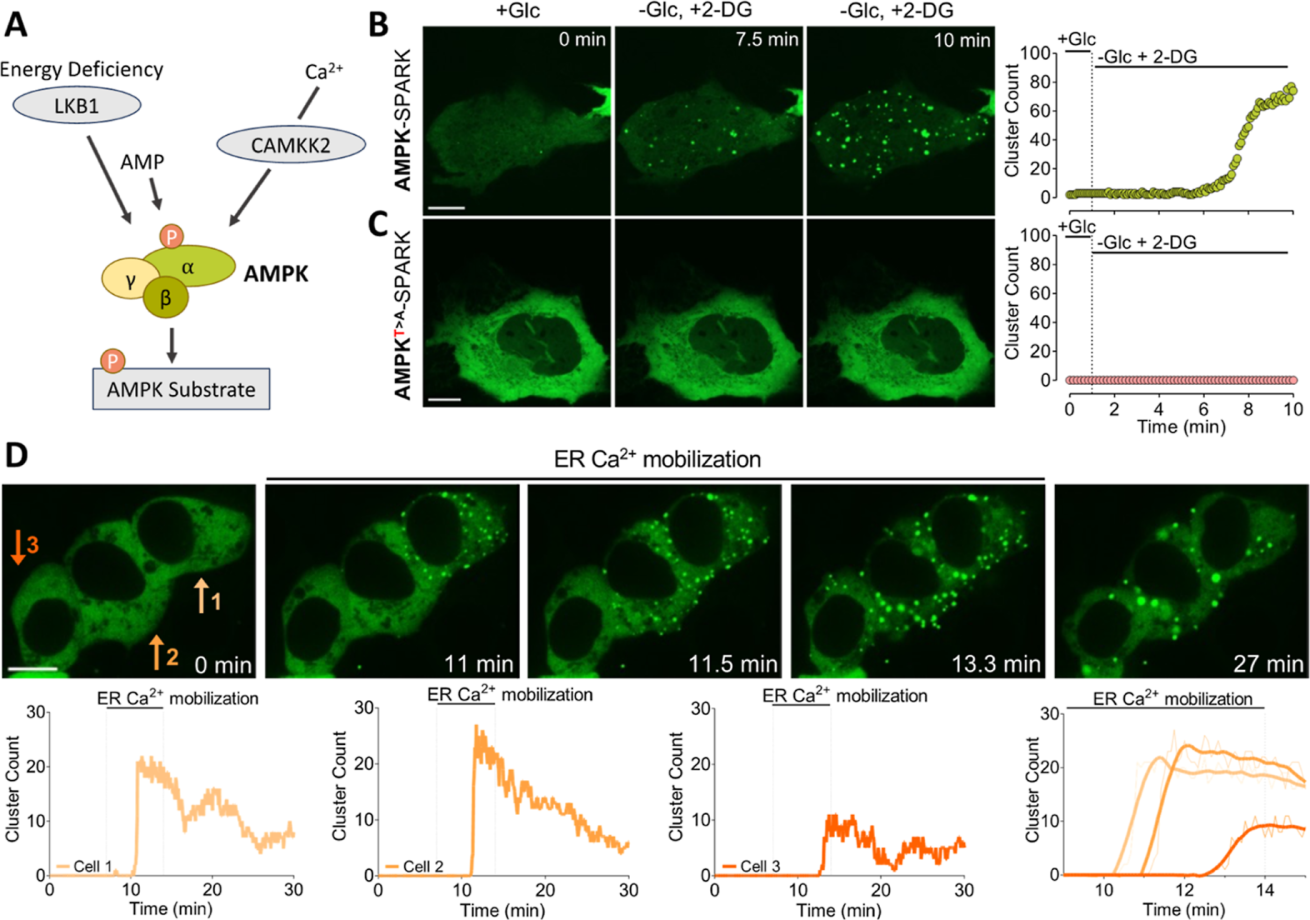
AMPK-SPARK dynamically forms clusters in response to various stresses. (A) Schematic shows the fundamental mechanisms for the activation of AMPK, which consists of 3 subunits (α, β, γ). The confocal images demonstrate (B) AMPK-SPARK or (C) phospho-null AMPK^*T*>A^-SPARK expressing single EA.hy926 cells at indicated time points and their respective time-course traces of cluster formation upon perfusion with glucose-free imaging buffer supplemented with 5 mM 2-deoxyglucose (2-DG). (D) Three adjacent HEK293D cells (cell 1, 2, 3) have been subjected to Ca^2+^ mobilization from ER achieved by the administration of 100 μM carbachol and 15 μM BHQ. The confocal images and the corresponding time-course traces of cluster count of the indicated cells are presented in the top and bottom rows, respectively. The last panel in the bottom row depicts the comparative cluster count of the cells with a close-up time scale. Scale bars are 10 μm. All images were taken at 5 s intervals, with no binning, in confocal mode.

The activation of AMPK occurs through distinct pathways: the canonical pathway, induced by changes in the ATP/ADP/AMP ratios, facilitated by the upstream kinase LKB1, and noncanonical pathways, including activation via elevated cytosolic Ca^2+^ levels, mediated by CaMKK2 (Figure 2A).^12^ To achieve rapid activation of endogenous AMPK activity, we thus subjected HEK293 cells to glucose deprivation to trigger energy stress while simultaneously stimulating them with a mixture of compounds (ATP and BHQ) that efficiently mobilize Ca^2+^ to elevate cytosolic Ca^2+^ levels. The combination of canonical and noncanonical routes of AMPK activation resulted in the swift formation (1.56 ± 0.30 min, 8 cells) of numerous AMPK-SPARK droplets in multiple cells (Supporting Information, Videos 1 and 2). In some cells, the stimulation led to droplets that gradually increased in size and persisted even after stimulation, while smaller droplets swiftly dissipated (Supporting Information, Videos 1 and 2). This observation aligns with our findings of AMPK-SPARK droplets of varying sizes across different cell types in the culture (Figure 1B,D). However, most droplets (86% ± 5.62 of counted clusters in 8 cells) dispersed upon glucose reintroduction and withdrawal of the Ca^2+^-mobilizing compounds, pointing to the fundamental reversibility of the AMPK-SPARK tool following transient stimulation of the AMPK pathway (Supporting Information, Videos 1 and 2). To quantitatively illustrate the dynamic AMPK activity over time, utilizing the novel AMPK-SPARK tool, throughout the study, we opted for X–Y diagrams plotting the number of droplets of a single cell against the time. Upon induction of energy stress through glucose removal and simultaneous elevation of cytosolic Ca^2+^ by depleting Ca^2+^ stored in the endoplasmic reticulum (ER), a substantial and rapid increase in the count of individual AMPK-SPARK droplets was observed (Figure S7A,B). Following an initial peak, the droplet counts stabilized, gradually decreasing only after the end of stimulation (Figure S7B). The initial peak observed in the AMPK-SPARK curve is due to the emergence of small clusters. The signal then dropped as clusters coalesced over time (Supporting Information, Video 1, the middle-very left cell). Cells expressing AMPKAR, a FRET-based AMPK reporter,^4^ immediately responded (1.07 ± 0.17 min, 7 cells) to the same strong stimulus. Overall, under identical conditions, we observed comparable readouts for the activation and the deactivation of endogenous AMPK in HEK293D cells between AMPKAR and AMPK-SPARK (Figure S7B,C). It should also be noted that while FRET-based or single FP-based AMPK reporters have a unimolecular design, AMPK-SPARK has a bimolecular design. Unimolecular biosensors go through a conformational change as fast as nanoseconds,^33^ whereas bimolecular biosensors can be slowed down due to diffusion.^34^ Moreover, to achieve a detectable signal, AMPK-SPARK needs to concentrate enough protein domains. Together, these all may delay the response time of the SPARK-based kinase biosensors^35^ and make AMPK-SPARK less sensitive than unimolecular AMPK reporters.

Axotomy is an in vitro neuronal injury experimental model system for investigating cellular responses involving significant fluctuations in cellular ATP and Ca^2+^ levels.^36,37^ To visualize AMPK activation during axotomy on the single-cell level, we introduced the AMPK-SPARK construct into freshly isolated primary mouse cortical neurons and monitored its behavior following axotomy (Figure S8). Out of 23 single axotomized neurons, 9 cells exhibited a clear AMPK-SPARK response that persisted throughout the observation period (Figure S8). It is noteworthy that not all neurons exhibited an AMPK-SPARK response to axonal injury, reflecting the inherent heterogeneity of neuronal responses. Collectively, these experiments underscore the applicability of SPARK technology for visualizing AMPK activation in neuronal cells.

Next, we treated endothelial cells with 2-DG, which induces robust energy stress. Following the substitution of glucose with 2-DG, a progressive elevation in the formation of AMPK-SPARK clusters was observed (Figure 2B), indicating gradual AMPK activation upon energy stress. Notably, no clusters emerged in 2-DG treated cells expressing the AMPK-insensitive variant AMPK^(*T*>A)^-SPARK (Figure 2C). These findings emphasize that SPARK-AMPK can also report the activation of AMPK solely by instigating energy stress through ATP reduction in the endothelial cell line. We then monitored the formation of AMPK-SPARK clusters induced exclusively via Ca^2+^ elevation by ER Ca^2+^ depletion in three adjacent HEK293 cells (Figure 2D). Despite the simultaneous treatment of the cells using a gravity-driven perfusion system with Ca^2+^-mobilizing compounds, the clusters did not emerge at the same time across the three cells; instead, they formed with noticeable temporal delays. These delays may imply variances in Ca^2+^ signal dynamics (including variances in amplitude, frequency, duration, delay, or cumulative level).^38^ Moreover, different cellular responses to the induction of AMPK activation may stem from different capacities of ATP production and turnover,^39^ elusive upstream mechanisms,^40^ or metabolic history of the single cells.^40^ Following Ca^2+^ mobilization, the number of clusters per cell declined significantly (Figure 2D and Supporting Information, Video 3), showing the reversibility of Ca^2+^-triggered AMPK activation. We hypothesize that the remaining clusters reflect a memory of the previous AMPK activation (Figure 2D and Supporting Information, Video 3).

### Design of a Dual Ca^2+^ AMPK-SPARK Reporter System Integrating GCaMP6f

**Figure 3:**
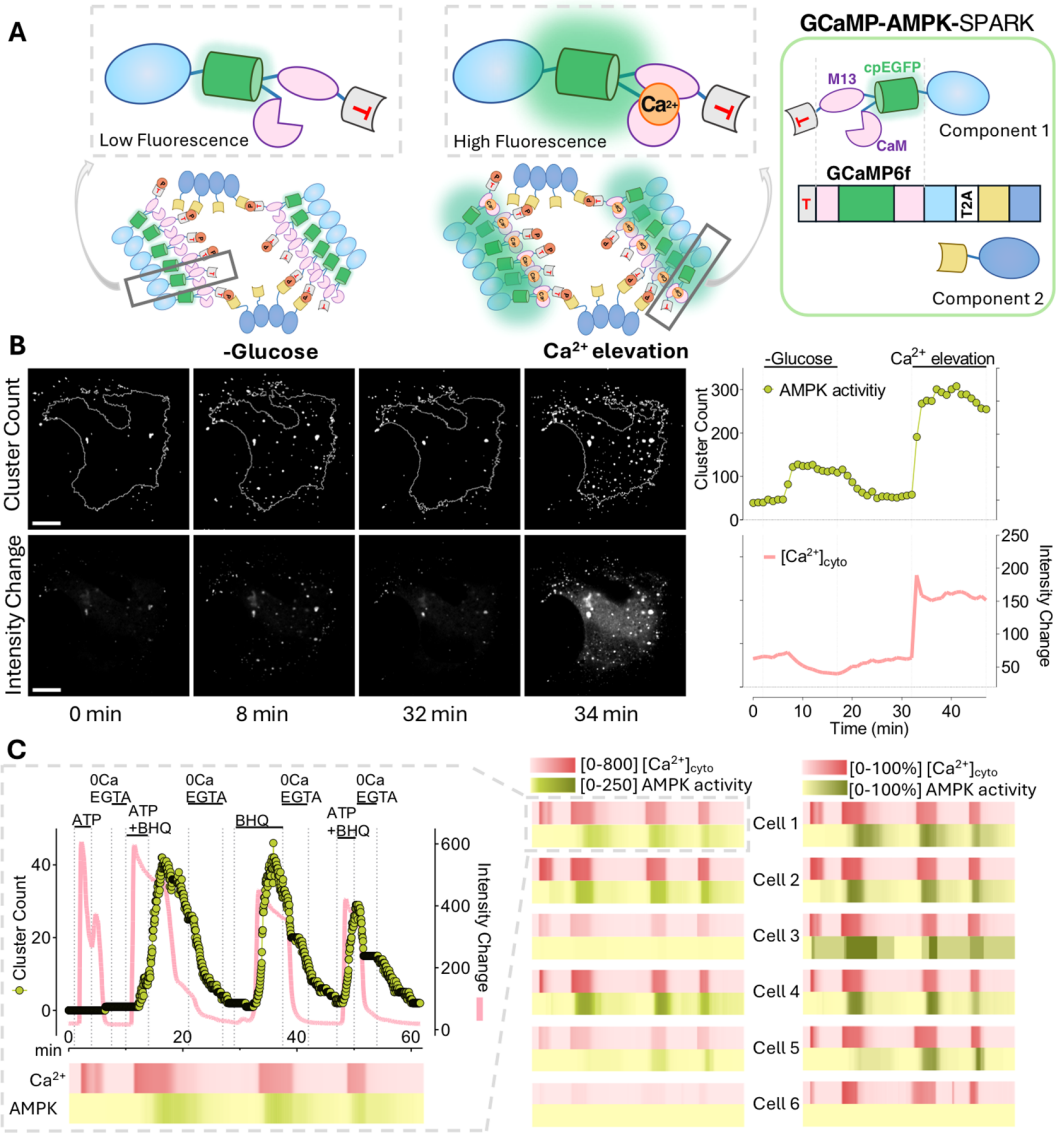
GCaMP-AMPK-SPARK reported variances in Ca^2+^-mediated AMPK activation. (A) Cartoons depict clustered GCaMP-AMPK-SPARK reporters under AMPK-activated conditions; glucose deprivation, and Ca^2+^ elevation. Single units of component 1 under these two conditions are zoomed in for a better comparison. The cartoon at the very right depicts the components of the GCaMP-AMPK-SPARK construct. (B) EA.hy926 cells transiently transfected to express GCaMP-AMPK-SPARK. The first (binarized images to emphasize cluster count) and second (raw images to emphasize intensity changes) rows of images show a single EA.hy926 cell that has been subjected to glucose deprivation, glucose reintroduction, and Ca^2+^ elevation by 100 μM ATP and 15 μM BHQ at indicated time points. Cell outline was defined using raw images. The top graph shows the time-course quantification of cluster count, reporting AMPK activity over time (curve with green circles). The red curve in the bottom graph shows intensity changes in integrated GCaMP6f, reporting changes in cytosolic Ca^2+^ levels. Images were taken at 1 min intervals with 2 × 2 binning in confocal mode. (C) The graph showcases the overall intensity change of this cell in pink and the respective changes in AMPK activity with cluster count in green circles. Cells are subjected to Ca^2+^ elevation by perfusion of different buffers (including 100 μM ATP, 15 μM BHQ, or a combination of the two) at designated time points. Cells are subjected to Ca^2+^ removal by the perfusion of 0 mM Ca^2+^ imaging buffer with 1 mM EGTA at indicated time points. Single-cell heatmaps illustrate the changes in GCaMP-AMPK-SPARK intensity, i.e., cytosolic Ca^2+^ signals, and cluster count, i.e., AMPK-activation, to the given protocol in the graph. Images were taken at 3 s intervals with no binning in confocal mode.

To shed light on Ca^2+^-mediated AMPK activity, we developed a dual reporter system that allows for simultaneous monitoring of intracellular Ca^2+^ levels and AMPK activity. To accomplish this, we replaced EGFP in AMPK-SPARK with a single FP-based Ca^2+^ biosensor, GCaMP6f. We named this new dual reversible reporter GCaMP-AMPK-SPARK, which enables the simultaneous monitoring of two parameters through a single GFP channel, providing a comprehensive view of Ca^2+^-coupled AMPK activation. While an increase in cytosolic fluorescence reflects a rise in intracellular Ca^2+^ levels, cluster formation reports an increase in AMPK activity (Figure 3A, Graphical Abstract).

To test the functionality of the dual reporter, we expressed GCaMP-AMPK-SPARK in the EA.hy926 cells. We observed cluster formation without intensity elevation upon glucose removal, indicating Ca^2+^-independent AMPK activation. With glucose reprovision, the clusters dissolved, indicating the reversibility of GCaMP-AMPK-SPARK. Then, we further stressed the cell by using Ca^2+^ mobilizing agents and observed a significant increase in the number of clusters that emerged (Figure 3B, top row and Supporting Information, Video 4) and in the fluorescence intensity of the cell (Figure 3B, bottom row and Supporting Information, Video 5), indicating a concurrent rise in AMPK activity and cytosolic Ca^2+^ levels, respectively. The reduction in intensity observed (Figure 3B, bottom row) during cluster formation is likely due to the accumulation of FP out of the focal plane.

### Visualizing Cell-to-Cell Heterogeneities of Ca^2+^-AMPK Coupling, Employing the Dual Reporter System

Next, we conducted simultaneous visualization of Ca^2+^ signals and AMPK activation of individual HeLa cells expressing the dual reporter system (Figure 3C). To induce varied Ca^2+^ signals, we treated cells sequentially with Ca^2+^-mobilizing compounds. Intriguingly, not all cytosolic Ca^2+^ signals led to a notable cluster formation. In Cell 1, initial ATP treatment did not induce cluster formation despite triggering a prominent Ca^2+^ elevation. Interestingly, the subsequent re-elevation of Ca^2+^ signals yielded conspicuous cluster formation, demonstrating discrepancies in Ca^2+^-coupled AMPK activation. We employed a color code to illustrate the outcomes of the dual reporter system in several cells from the same imaging region (Figure 3C, heatmaps). In all cells imaged, we observed similar temporal patterns of Ca^2+^ elevation upon administration of Ca^2+^ mobilizing agents, yet not all of these signals have translated into a parallel increase in AMPK activity (Figure 3C, Supporting Information, Video 6). Notably, the cluster formation of Cell 6 may not be determined due to its low expression of GCaMP-AMPK-SPARK. These experiments imply a cell-to-cell heterogeneity related to the implications of Ca^2+^ signals for AMPK activation. Additionally, we detected a similar variance in Ca^2+^-coupled AMPK activation using the Ca^2+^ ionophore, ionomycin (Figure S9). With ionomycin, intracellular Ca^2+^ levels are elevated to supraphysiological levels, minimizing the potential variances in Ca^2+^ signals.^38^ Under these conditions, we observed adjacent cells exhibiting apparent differences in the temporal linkage between the Ca^2+^ signal and cluster formation, indicating heterogeneities in AMPK activation (Figure S9B,C). The heterogeneity among single cells is a common attribute of dynamic metabolic processes,^41,42^ which has been reported for various aspects of subcellular AMPK activity as well as for other kinases.^5,7−9,39,40,43−45^ Our results with cell lines and primary cultures of cortical neurons add to the expanding body of evidence supporting the metabolic heterogeneity at the single-cell level.

## Conclusions

The new phase-separation-based AMPK activity reporter, AMPK-SPARK, expands the toolkit of researchers who study AMPK signaling. This reporter works reversibly in a single fluorescent channel with a high signal-to-noise ratio. The high dynamic range in terms of fluorescent change amplifies the signal, making AMPK-SPARK less prone to artifacts, such as autofluorescence or light scattering effects. This makes AMPK-SPARK suitable for high-throughput drug screening and tissue applications. The simple signal pattern of SPARK-based sensors provides a clear and direct qualitative readout of kinase activity. On the other hand, while FRET-based or single FP-based AMPK biosensors are quantitative, AMPK-SPARK rather falls semiquantitative. AMPK-SPARK relies on the qualitative imaging of single cells, which restricts its quantitative use to heterogeneous cell populations. To quantify the relative status of AMPK activity between single cells using the SPARK constructs, one could choose to calculate the sum of the fluorescence intensity of clusters over the whole cell intensity,^9,15,16^ the cluster count,^35^ or size^46^ or define cells based on the size of SPARK clusters. Moreover, the very small AMPK-SPARK clusters that lack sufficient contrast (against the nearby nonclustered fluorescence) cannot be determined. Therefore, the response of AMPK-SPARK biosensors may be delayed by protein recruitment, rendering the sensors less sensitive to minor changes in AMPK activity or when the sensor has low levels of expression. Furthermore, it should be noted that AMPK-SPARK lacks the high spatial resolution of targeted unimolecular AMPK biosensors.

The GCaMP-AMPK-SPARK combines SPARK technology with a single FP-based Ca^2+^ biosensor, demonstrating a proof of concept of a new dual reporter system. GCaMP-AMPK-SPARK can report Ca^2+^ levels, providing a comprehensive look into Ca^2+^-mediated AMPK activity. The dual reporter system is a refined method to monitor an intracellular messenger (i.e., Ca^2+^) and kinase activity (i.e., AMPK) in a single fluorescence channel, at the same time.

Imaging with GCaMP-AMPK-SPARK is thus advantageous, as it yields two layers of information simultaneously. However, integrating GCaMP6f, which has very large changes in fluorescence upon Ca^2+^ elevations, complicates cluster analysis based on intensities. For this reason, we opted for cluster counting throughout the study. While cluster counting can report the relative changes in AMPK activity in dynamic measurements, we acknowledge that it lacks the cluster size metrics, as well as the reciprocal information on nonclustered components in the cytosol. Although we intentionally utilized only one fluorescence channel in this study, our dual reporter approach, merging intensiometric single FP-based biosensors with SPARK technology, can be expanded to include additional colors. We anticipate that this expansion will enable the visualization of several signaling processes and their corresponding kinase activities in a multiplexing mode.

## Experimental Section

### Buffers and Solutions

Cell culture materials were obtained from Greiner Bio-One (Kremsmünster, Austria). During z-stack imaging and before time-course imaging, cells were equilibrated and incubated in physiological storage solution (in mM): 2 CaCl_2_, 135 NaCl, 1 MgCl_2_, 5 KCl, 10 HEPES, 2.6 NaHCO_3_, 0.44 KH_2_PO_4_, 0.34 Na_2_HPO_4_, 1x amino acids, 1x vitamins, 10 glucose, and 2 l-glutamine with a pH of 7.45. The physiological imaging buffer used during time-course microscopy experiments contains (in mM): 138 NaCl, 5 KCl, 2 CaCl_2,_ 1 MgCl_2_, and 10 d-glucose, pH adjusted to 7.4 with NaOH. The modifications on the imaging buffer are indicated in the figures as well as in the figure legends. The Ca^2+^-free imaging buffer contains (in mM): 138 NaCl, 5 KCl, 1 EGTA, 1 MgCl_2_, and 10 d-glucose, pH adjusted to 7.4 with NaOH. Adenosine 5′-triphosphate disodium salt (ATP) was purchased from Carl Roth (Graz, Austria). 2,5-di(*tert*-butyl)-1,4-hydroquinone (BHQ) was purchased from Sigma-Aldrich (Massachusetts, United States). 2-Deoxy-d-glucose (2-DG) was purchased from Thermo Fisher Scientific (Massachusetts, United States).

### Construct Design

The SPARK-AMPK was generated by the substitution of kinase substrate sequence in the previously designed SPARK-PKA biosensor.^9^ From the *N*-terminus, SPARK-AMPK insert consists of AMPK substrate peptide,^4^ EGFP, the homohexameric coiled coil HOTag3 domain, self-cleavage sequence (T2A), phosphothreonine-binding forkhead-associated domain 1 (FHA1), and the homotetrameric coiled HOTag6 domain. For GCaMP-AMPK-SPARK, EGFP in SPARK-AMPK was replaced with GCaMP6f.^14^ For AMPK^(*T*>A)^-SPARK, the phospho-threonine within the AMPK substrate sequence was replaced with an alanine. Inserts were synthesized and cloned into the pTwist CMV backbone by Twist Biosciences (California, United States).

### Cell Culture and Transfection

HEK 293D, HeLa, and EA.hy926 cells were cultured in Dulbecco’s modified Eagle’s medium (DMEM D5523, Sigma-Aldrich) supplemented with 10% FCS, 10 mM sodium bicarbonate, 50 U/mL penicillin–streptomycin, 1.25 μg/mL amphotericin B, and 25 mM HEPES; pH was calibrated to 7.45 with NaOH. Cells were grown in a humified incubator (5% CO_2_, 37 °C). Cells were seeded on 30 mm glass coverslips (Co. KG, Lauda-Königshofen) in 6-well plates (Paul Marienfeld GmbH, Germany). For transient transfection, a transfection reagent PolyJet (SignaGen Laboratories, Rockville, USA) was used according to the manufacturer’s instructions. Live-cell imaging was performed 24–48 h after transfection.

For investigating the Mg^2+^ effect on cellular energy status, EA.hy926 cells were incubated in modified custom media (0MgCl_2_, 0NaCl) by Cytvia (Marlborough, United States) supplemented with 10% FCS, 10 mM sodium bicarbonate, 50 U/mL penicillin–streptomycin, 1.25 μg/mL amphotericin B, and 25 mM HEPES; pH was calibrated to 7.45 with NaOH. For different concentrations of MgCl_2_, NaCl levels were modified accordingly to maintain isoosmotic solutions.

Primary neuron cultures were established following previously described protocols and slightly adapted in this study to cortical neuron cultures.^36^ Mice at the postnatal stage of p3 were humanely euthanized by using CO_2_, and their brains were carefully isolated. The cortex was dissected and sliced into small pieces under a stereo microscope (Zeiss Discovery V8) in a solution containing 1% antibiotic-antimycotic (Sigma, A5955) and L15 medium (ThermoFisher Scientific, A1247501). Tissue digestion was carried out with papain (Sigma, P4762) at 4 °C for 45 min. Subsequently, DNase (Sigma, D5025) was introduced, and the tissues were homogenized through serial pipetting. The enzymatic activity was halted by adding 10% FBS at 4 °C for 15 min, and the medium was removed via centrifugation at 180*g* for 5 min. The isolated cells were resuspended in Neurobasal-A (Gibco, 10888022) supplemented with 1% antibiotic-antimycotic solution, 1% GlutaMAX (Gibco, 35050061), and 2% B27 (Gibco, 17504044). These cells were then plated on glass-bottom dishes coated with poly-d-lysine and maintained at 37 °C with 5% CO2. Following a 48 h incubation period, cells were transfected using lipofectamine (Invitrogen, 11668019) at a plasmid-to-lipofectamine ratio of 1:3, following the manufacturer’s instructions.

### Animal Experiments

The animal experiments conducted in this study received approval from the Istanbul Medipol University Animal Experimentation Ethical Committee. All procedures were performed in strict accordance and full compliance with European Council Directive 2010/63/EU.

### Live Cell Imaging

Before imaging, cells were preincubated in a physiological storage solution for at least 20 min at room temperature. For high-resolution live cell imaging, an array confocal laser scanning microscope (Axiovert 200 M, Zeiss), equipped with a 100×/1.45 NA oil immersion objective (Plan-Fluor, Zeiss) and a Nipkow-based confocal scanner unit (CSU-X1, Yokogawa Electric Corporation), was used. The fluorescent proteins within the biosensors were illuminated with an F488 diode laser (Visitron Systems, Puchheim, Germany). Emission light was collected with a CoolSNAP HQ2 CCD Camera (Photometrics Tucson, Arizona, USA) using the ET525/36m emission filter (Chroma Technology Corporation). Alternatively, a Nikon Eclipse Ti2 microscope was used for time-lapse imaging. The microscope was equipped with a 40 ×/1.15 NA water immersion objective (CFI Apochromat, Nikon), 100*×*/1.45 NA oil objective (CFI Apochromat, Nikon) standard filter sets, and two Kinetix Scientific CMOS cameras (Photometrics). Excitation of reporters was achieved with 476 nm laser light (Celesta, Light Engine) and emission was collected at 501–521 nm.

For FRET-imaging of AMPKAR, we employed an NGFI AnglerFish C–Y7G widefield microscope equipped with a 40× oil immersion objective (Plan Apochromat 1,3 NA Oil DIC (UV) VISIR, Carl Zeiss GmbH, Vienna, Austria) and a standard CFP/YFP filter cube. The biosensor was excited at 440 nm (440AF21, Omega Optical, Brattleboro, VT, USA), and emissions were collected at 480 and 535 nm (480AF30 and 535AF26, Omega Optical, Brattleboro, VT, USA) using a 505 dcxr beam splitter on two sides of the camera (CCD camera, CoolSNAP Dyno, Photometrics, Tucson, AZ, USA).

During the time-course measurements, cells were continuously perfused via a gravity-based perfusion system (NGFI, Austria).

For laser axotomy, neurons were preincubated in a physiological salt solution (pH 7.42) for 1 h at room temperature before imaging. Continuous time-lapse images were acquired using a confocal microscope (Zeiss LSM 780) equipped with a 40*x*/1.4 oil objective and a 488 nm excitation laser. The detector was tuned to capture emissions ranging from 493 to 598 nm, and the pixel dwell time was set at 1.27 μs. Laser axotomy was performed during the time-lapse imaging using a femtosecond laser system (Coherent) tuned to 790 nm with a 690+ MBS filter. The laser was operated at 100% power with 3 iterations.

### Image Analysis

For cluster quantification, images were processed in ImageJ. For AMPK-SPARK, the stack of time-lapsed images was analyzed using a macro script to run the “Find Maxima/Count” function by manual thresholding.

GCaMP-AMPK-SPARK cluster quantification was done by using the same function. Here, two concurrent thresholding were done; a high threshold to capture clusters that emerged under increased Ca^2+^ levels and a lower threshold to capture clusters when Ca^2+^ levels are low. These two data points were then merged manually.

For analyses in Figure S3, confocal images were background subtracted using a background ROI. Using the corrected z-stack, the mean intensity of each cell was determined. To evaluate the cluster count and morphology, the original z-stacks were background corrected using a rolling ball background subtraction. To increase the contrast and simplify segmentation, the background corrected image was pixel-wise multiplicated with itself and subsequently scaled down to an 8-bit format. A Triangle auto threshold using the z-stack intensity histogram was applied to segment single clusters. The Fiji plugins 3D Volume, 3D Compactness, and 3D Ellipsoid were used to measure cluster count, cluster volume, and cluster sphericity, respectively.

For Figure 3B and Supporting Information,Video 4, images were subjected to background subtraction with a rolling stack and each frame multiplied with itself to create a 32-bit stack. Then, the minimum and maximum displayed values were set to each frame’s mean and maximal fluorescence, respectively. Images were converted to 16 bit. Autothresholding with the Max Entropy function was applied to the stack images. The stack was then inverted. The Analyze Particles function was then applied to obtain the mask with clusters. The outline of the cell was generated using raw cell images through the Binary/Outline function. The cluster mask and outline mask were then merged to make the cellular boundary apparent.

### Data and Statistical Analysis

The data obtained were analyzed via Excel (Microsoft), MetaMorph (Molecular devices), and GraphPad Prism 5 Software (GraphPad Software, Inc., La Jolla, CA, USA).

To depict the single-cell heatmaps of AMPK activity and cytosolic Ca^2+^ levels in Figure 3D, we used the Conditional Formatting function in Excel. The manually corrected cluster count data and background subtracted fluorescence intensity data from image analysis were used for the first set of heatmaps (Figure 3D, left). For the second set of heatmaps (Figure 3D, right), we normalized the data to the maximal cluster count and maximal fluorescence of the individual cell.

The number of independent experiments is given as ‘*X*’, and the number of single cells is ‘*x*’. Each figure legend contains this statistical information as *n* = *X*/*x*. For instance, *n* = 3/120 indicates 3 biological replicates and a total of 120 cells that were analyzed.

## Supporting Information Available

Mapping endogenous PKA activity in different cell types using the SPARK technology; phospho-null mutant of AMPK-SPARK not showing any clusters upon its expression; expression level of AMPK-SPARK not correlating with cluster count or sphericity; elevated extracellular Mg^2+^ levels show higher AMPK activity than reduced extracellular Mg^2+^ levels; cluster morphology difference between AMPK-SPARK and PKA-SPARK; clusters of AMPK-SPARK and PKA-SPARK with different spatial arrangements; AMPK-SPARK and FRET-biosensor AMPKAR show analogous readouts; primary cortical neurons exhibit heterogeneous AMPK responses to axotomy injury; and dual reporter unveils variances in Ca^2+^-mediated AMPK activation under supraphysiological Ca^2+^ levels (PDF)
Canonical and noncanonical activation of AMPK in HEK293 cells expressing AMPK-SPARK (Position 1) (AVI)
Canonical and noncanonical activation of AMPK in HEK293 cells expressing AMPK-SPARK (Position 2); (AVI)
Ca^2+^ elevation mediated AMPK activation in HEK293 cells expressing AMPK-SPARK (AVI)
Cluster emergence and dissolution upon glucose removal, glucose reintroduction, and Ca^2+^ elevation in a EA.hy926 cell expressing GCaMP-AMPK-SPARK (AVI)
Intensity changes upon glucose removal, glucose reintroduction, and Ca2+ elevation in an EA.hy926 cell expressing GCaMP-AMPK-SPARK (AVI)
HeLa cells expressing GCaMP-AMPK-SPARK in response to sequential increase and reduction of cytosolic Ca^2+^ (MP4)

## Supplementary Material

se4c01058_si_001.pdf

se4c01058_si_002.avi

se4c01058_si_003.avi

se4c01058_si_004.avi

se4c01058_si_005.avi

se4c01058_si_006.avi
