# Supplementary material for: Development of a Dual Reporter System to Simultaneously Visualize Ca2+ Signals and AMPK Activity
Source: ACS Sens. 2024 Aug 21;9(9):4680–9. doi: 10.1021/acssensors.4c01058 (PMC11443530; doi:10.1021/acssensors.4c01058)
Supplement: Supplementary file 1 — se4c01058_si_001.pdf [file se4c01058_si_001.pdf]

Supplementary Information to

Development of a Dual Reporter System to Simultaneously Visualize Ca^{2+} Signals and AMPK activity

Yusuf C. Erdogan[1,2], Johannes Pilic[1], Benjamin Gottschalk[1], Esra N. Yiğit[3,4], Asal G. Zaki[3], Gürkan Öztürk[3], Emrah Eroglu[3], Begüm Okutan[5], Nicole G. Sommer[5], Annelie M. Weinberg[5], Rainer Schindl[6], Wolfgang F. Graier[1,2], and Roland Malli[2,7*]

[1]Gottfried Schatz Research Center, Molecular Biology and Biochemistry, Medical University of Graz, Neue Stiftingtalstraße 6, 8010, Graz, Austria
[2]BioTechMed Graz, Mozartgasse 12/2, 8010, Graz, Austria
[3]Regenerative and Restorative Medicine Research Center (REMER), Research Institute for Health Sciences and Technologies (SABITA), Istanbul Medipol University, Istanbul 34810, Türkiye
[4]Department of Physiology, International School of Medicine, İstanbul Medipol University, İstanbul, 34810, Türkiye
[5]Department of Orthopedics and Traumatology, Medical University of Graz, Auenbruggerplatz 5, 8036 Graz, Austria
[6]Gottfried Schatz Research Center, Biophysics, Medical University of Graz, Neue Stiftingtalstrasse 6, 8010 Graz, Austria
[7]Center for Medical Research, Bioimaging, Medial University of Graz, Neue Stiftingtalstrasse 6, 8010 Graz, Austria

*Lead contact and correspondence: roland.malli@medunigraz.at

Figure S1: Mapping endogenous PKA activity in different cell types using the SPARK technology
Figure S2: Phospho-null mutant of AMPK-SPARK does not show any clusters upon its expression
Figure S3: Expression level of AMPK-SPARK does not correlate with cluster count or sphericity, yet shows a moderate correlation with cluster volume in cells under energy stress
Figure S4: Elevated extracellular Mg^{2+} levels show higher AMPK activity than reduced extracellular Mg^{2+} levels
Figure S5: Cluster morphology differs between AMPK-SPARK and PKA-SPARK
Figure S6: The clusters of AMPK-SPARK and PKA-SPARK appear with different spatial arrangements
Figure S7: AMPK-SPARK and FRET-biosensor AMPKAR show analogous readouts
Figure S8: Primary cortical neurons exhibit heterogenous AMPK responses to axotomy injury
Figure S9: The dual reporter unveils variances in Ca^{2+}-mediated AMPK activation under supraphysiological Ca^{2+} levels

Supplementary Video 1: Canonical and non-canonical activation of AMPK in HEK293 cells expressing AMPK-SPARK (Position 1)
Supplementary Video 2: Canonical and non-canonical activation of AMPK in HEK293 cells expressing AMPK-SPARK (Position 2)
Supplementary Video 3: Ca^{2+} elevation mediated AMPK activation in HEK293 cells expressing AMPK-SPARK
Supplementary Video 4: Cluster emergence & dissolution upon glucose removal, glucose reintroduction and Ca^{2+} elevation in a EA.hy926 cell expressing GCaMP-AMPK-SPARK
Supplementary Video 5: Intensity changes upon glucose removal, glucose reintroduction, and Ca^{2+} elevation in an EA.hy926 cell expressing GCaMP-AMPK-SPARK
Supplementary Video 6: HeLa cells expressing GCaMP-AMPK-SPARK in response to sequential increase and reduction of cytosolic Ca^{2+}

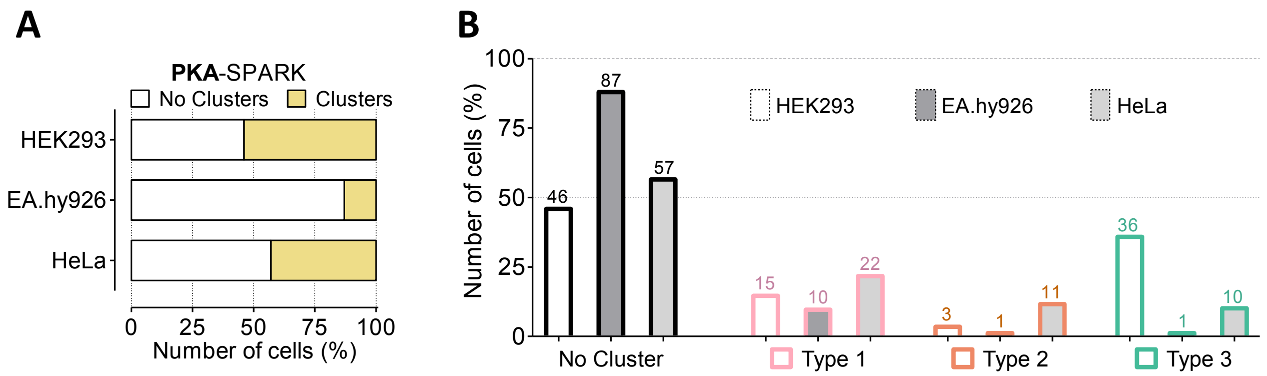

Figure S1. Mapping endogenous PKA activity in different cell types using the SPARK technology
A. Bar graphs represent the proportion of cells for the presence of clusters in HEK293d (n=3/198), EA.hy926 (n=4/83), and HeLa (n=3/69) upon transient expression of PKA-SPARK. **B.** The graph displays the type distribution in respective to cells described in panel A.

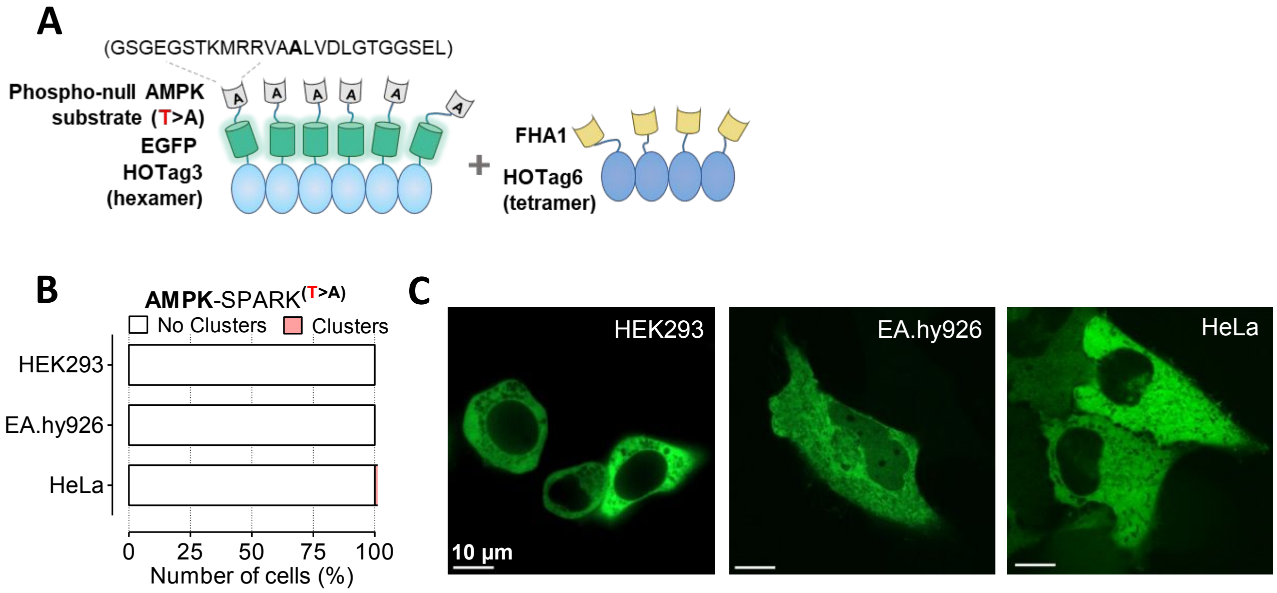

Figure S2. Phospho-null mutant of AMPK-SPARK does not show any clusters upon its expression
A. The cartoons show components of AMPK-SPARK$^{(T>A)}$. The phospho-threonine in the AMPK substrate sequence has been replaced with alanine to render the reporter insensitive to AMPK activity. **B.** Bar graphs represent the proportion of cells for the presence of clusters in HEK293d (n=2/112), EA.hy926 (n=4/93), and HeLa (n=3/127) upon transient expression of AMPK-SPARK$^{(T>A)}$. **C.** The pseudocolored confocal images represent cells expressing AMPK-SPARK$^{(T>A)}$.

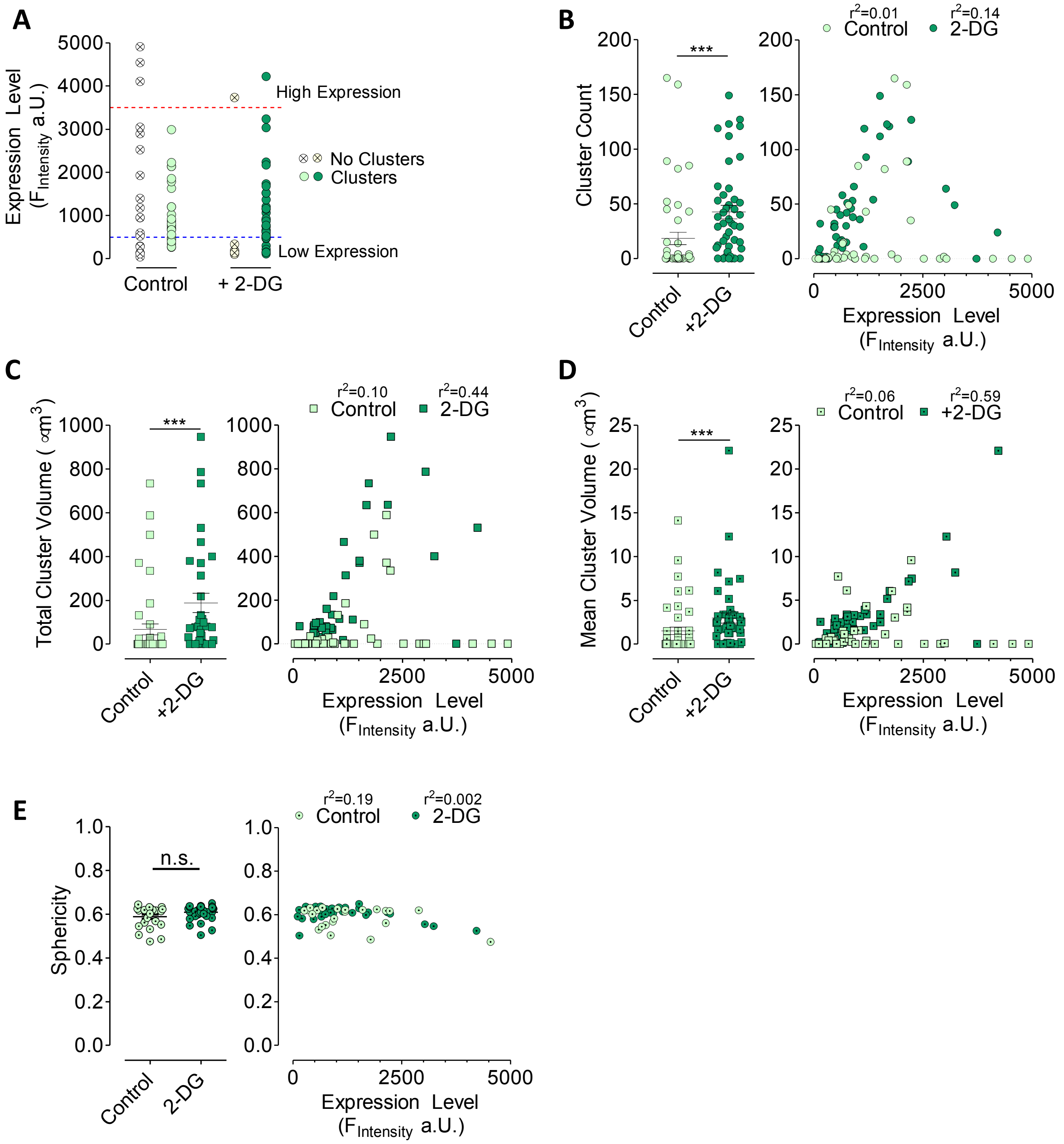

Figure S3. Expression level of AMPK-SPARK does not correlate with cluster count or sphericity, yet shows a moderate correlation with cluster volume in cells under energy stress.

For the comparative analyses, HeLa cells were transiently transfected with AMPK-SPARK, and high-resolution volumetric z-scan imaging was performed two days post-transfection, with z-stack spacing of 2 μm between each optical section. Cells were either untreated (n=3/46) or treated with glucose-free media supplemented with 20 mM 2-DG (n=4/43) for 9 hours prior to imaging. The scatter plot in **A.** demonstrates the distribution of cells with or without clusters based on their expression levels for the control or energy-stressed conditions. Scatter dot plots show the distribution of **B.** cluster count, **C.** total cluster volume, **D.** mean cluster volume, or **E.** sphericity distribution of individual cells under control or energy-stressed conditions or in relation to varying expression levels. Light green or dark green symbols represent the cells under control or energy-stressed conditions, respectively. The correlation coefficients were calculated by

performing linear regression analysis for each condition; the respective r^2 values are indicated above each legend. Statistical significance between groups was assessed using the non-parametric Mann-Whitney test. The plot indicates the mean ± SEM for each condition. The three asterisks indicate ***$p < 0.001$.

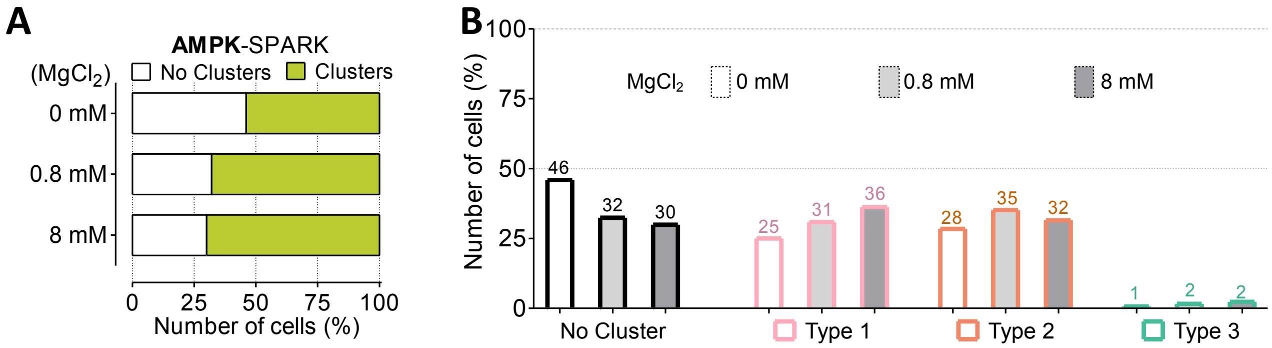

Figure S4. Elevated extracellular Mg^{2+} levels show higher AMPK activity than reduced extracellular Mg^{2+} levels

A. Bar graphs represent the proportion of cells for the presence of clusters in EA.hy926 ($MgCl_2$;0 mM, n=3/148: 0.8 mM, n=3/185 : 8 mM, n=3/127) cells upon transient expression of AMPK-SPARK. **B.** Type distribution in respective to cells described in panel A. The media with differential Mg^{2+} concentrations were prepared by modifying a custom media (0 mM $MgCl_2$ & 0 mM NaCl).

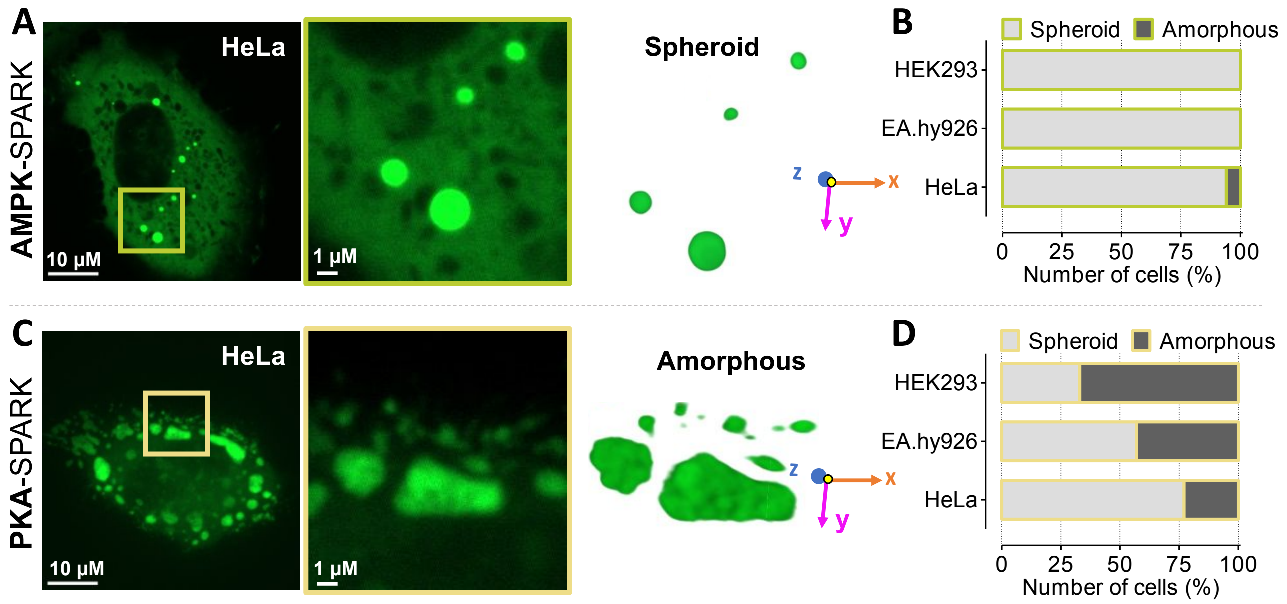

Figure S5. Cluster morphology differs between AMPK-SPARK and PKA-SPARK

A. The confocal images show a representative HeLa cell transiently expressing AMPK-SPARK. The middle panel shows a zoomed-in section from the cell. The third panel shows the 3D reconstitution of clusters from the z-stack images of the highlighted section. **B.** The bar graph shows the proportion of cells for the morphology of the clusters (spheroid vs amorphous) in HEK293d (n=3/209), EA.hy926 (n=2/49), HeLa (n=3/59) cells, transiently expressing AMPK-SPARK. **C.** The confocal images show a representative HeLa

cell transiently expressing PKA-SPARK. The middle panel shows a zoomed-in section from the cell. The third panel shows the 3D reconstitution of clusters from the z-stack images of the highlighted section. **D.** The bar graph shows the proportion of cells for the morphology of the clusters (spheroid vs amorphous) in HEK293d (n=3/107), EA.hy926 (n=2/14), HeLa (n=3/30) cells, transiently expressing PKA-SPARK.

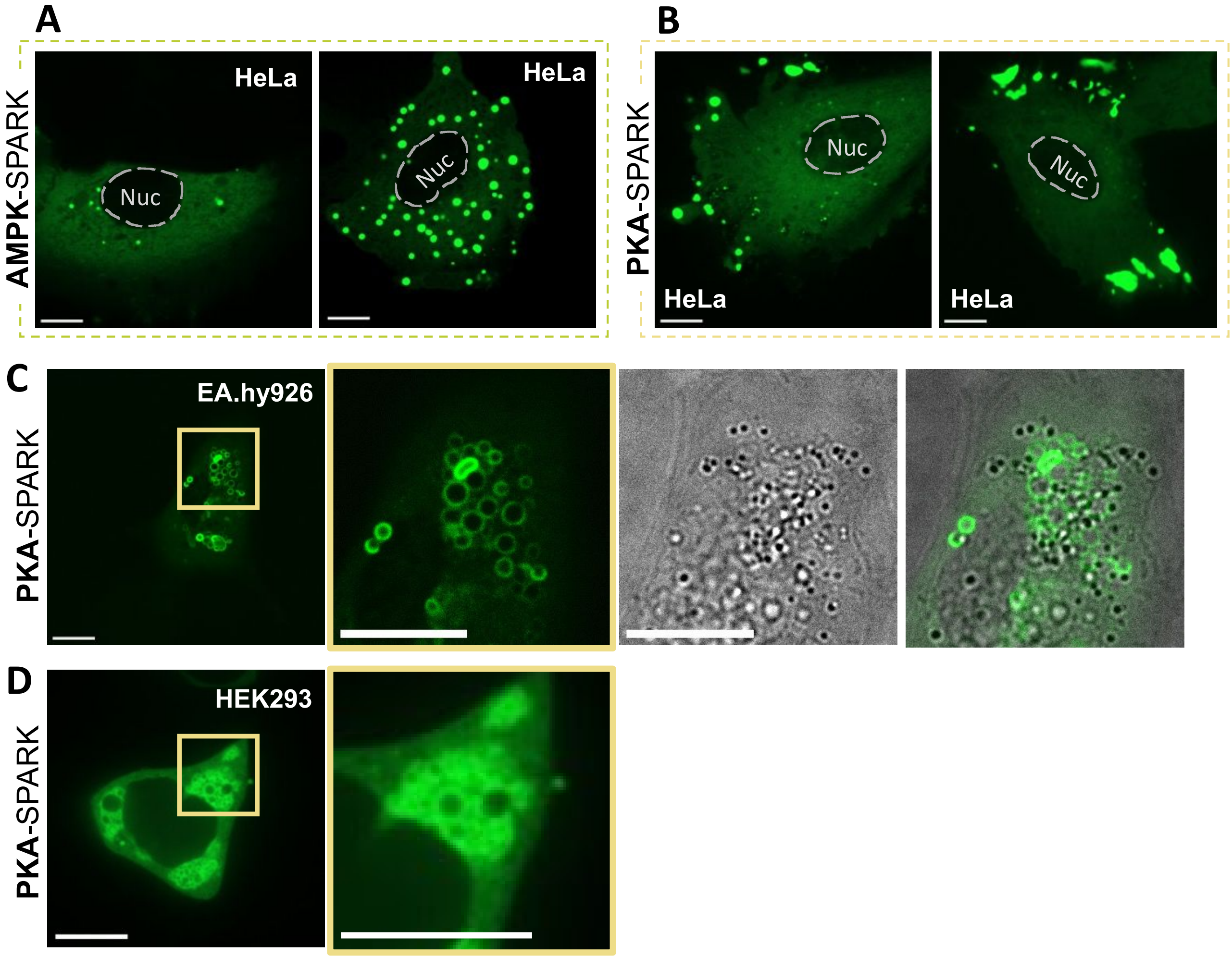

Figure S6. The clusters of AMPK-SPARK and PKA-SPARK appear with different spatial arrangements Images represent the spatial arrangement of **A.** AMPK-SPARK and **B.** PKA-SPARK clusters upon their transient expression in HeLa cells. The dashed line circling "Nuc" estimates the boundaries of the nuclear membrane. **C.** The panels show in order from left to right the following confocal images: a single EA.hy926 cell transiently expressing PKA-SPARK, the zoomed-in section from the cell, the brightfield image, and the merged image. **D.** The confocal images show a representative HEK293d cell transiently expressing AMPK-SPARK. The right panel shows the zoomed-in section from the left panel. All scale bars represent 10 μm.

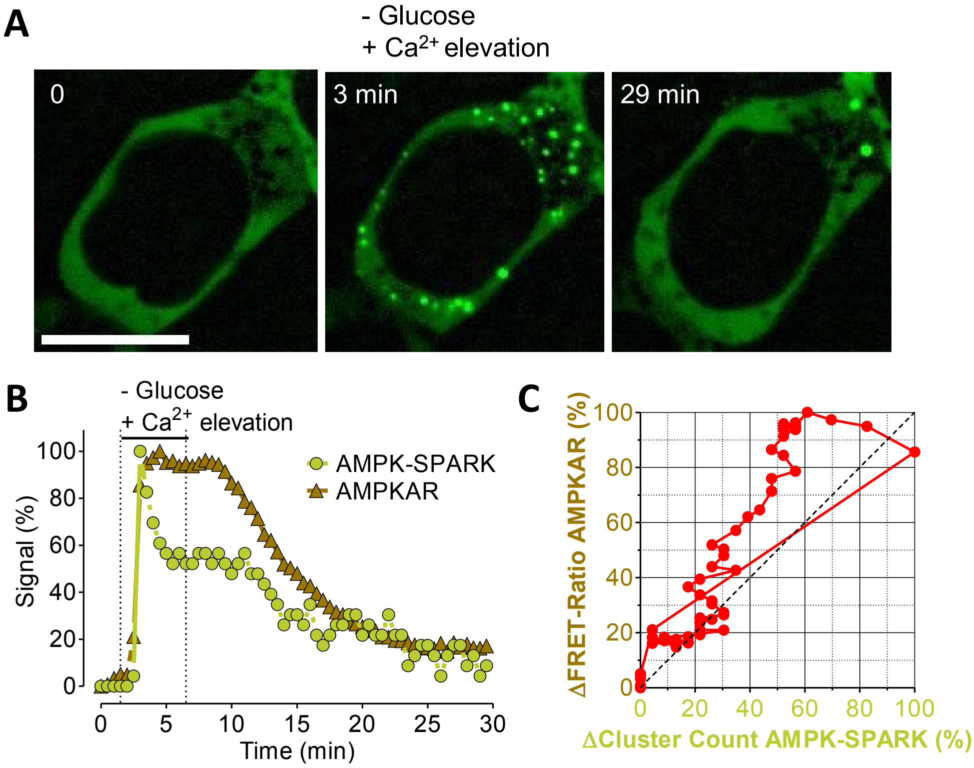

Figure S7. AMPK-SPARK and FRET-biosensor AMPKAR show analogous readouts

A. The confocal images show time-course activation of AMPK in response to glucose removal and concurrent Ca^{2+} elevation by administration of 100 μM ATP and 15 μM BHQ in a single AMPK-SPARK expressing HEK293d cell. The scale bar represents 10 μm. **B.** The curves with green circles and brown triangles represent single AMPK-SPARK or AMPKAR expressing HEK293D cells, respectively; in response to glucose removal and concurrent Ca^{2+} elevation by administration of 100 μM ATP and 15 μM BHQ. The signals are normalized to the maximal cluster count for AMPK-SPARK and the maximal FRET ratio for AMPKAR. For FRET measurement of AMPKAR, a widefield fluorescence microscope was employed. **C.** The correlative analysis of normalized optical readouts of AMPKAR and AMPK-SPARK is plotted.

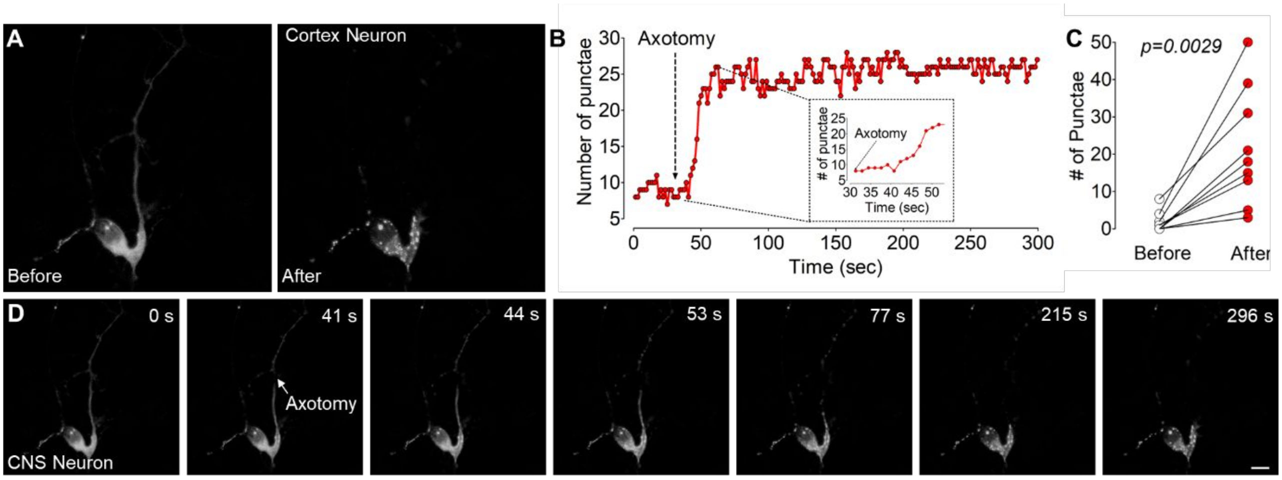

Figure S8. Primary cortical neurons exhibit heterogenous AMPK responses to axotomy injury
A. The representative confocal images of transiently SPARK-AMPK expressing mouse primary cortical neurons, before injury (left panel) and after axotomy (right panel). **B.** The representative real-time traces of cluster (punctae) formation over time in a single neuron (panel A) upon axotomy. The inset shows a close-up of the time scale of cluster formation and stabilization. **C.** Symbol and lines plot shows the numbers of clusters in different neurons before (white circles) and after (red circles, n=3/29) axotomy. **D.** The confocal images represent the time course of cluster formation in a single neuron. The paired Student's test was applied for the statistical evaluation of panel C. The scale bar represents 10 μm.

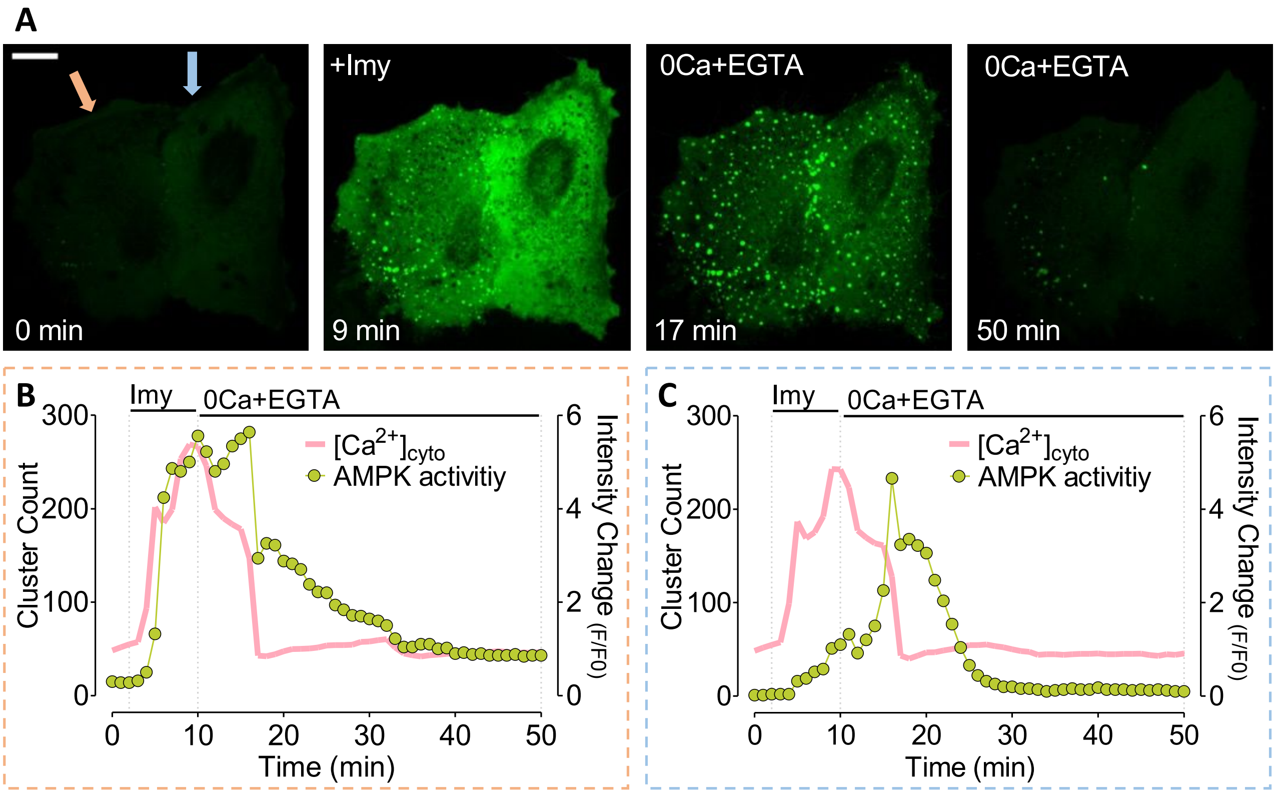

Figure S9. The dual reporter unveils variances in Ca^{2+}-mediated AMPK activation under supraphysiological Ca^{2+} levels

A. The confocal images show time-course activation and deactivation of AMPK in GCaMP-SPARK-AMPK expressing HeLa cells. Cells are subjected to Ca^{2+} elevation by administration of 1 μM ionomycin (Imy), and removal of Ca^{2+} by perfusion of 0 mM Ca^{2+} imaging buffer with 1 mM EGTA, respectively. The orange and blue arrow indicate the cell on the left and right, respectively. The graphs for the cell on the left (**B.**) and right (**C.**) showcase the overall intensity change in pink, and the respective changes in AMPK activity with cluster count in green circles. Images were taken in one-minute intervals in confocal mode.
